# Processing and Study of Optical and Electrical Properties of (Mg, Al) Co-Doped ZnO Thin Films Prepared by RF Magnetron Sputtering for Photovoltaic Application

**DOI:** 10.3390/ma13092146

**Published:** 2020-05-06

**Authors:** Chayma Abed, Susana Fernández, Selma Aouida, Habib Elhouichet, Fernando Priego, Yolanda Castro, M. B. Gómez-Mancebo, Carmen Munuera

**Affiliations:** 1 Department of Physics, University of Tunis El Manar, 2092 Tunis, Tunisia; abedchayma89@gmail.com; 2 Energy Department, CIEMAT, 28040 Madrid, Spain; 3 Laboratoire de Photovoltaïque, Centre de Recherches et des Technologies de l’Energie, BP 95, Technopôle de Borj-Cedria, 2050 Hammam-Lif, Tunis, Tunisia; saouida2002@yahoo.fr; 4 Physics Department, College of Sciences, University of Bisha, Saudi Arabia, P.B. 551, Bisha 61922, Saudi Arabia; habib.elhouichet@gmail.com; 5 Laboratoire de Caractérisations, Applications et Modélisation des Matériaux LR18ES08, Sciences Faculty of Tunis, University of Tunis El Manar, 2092 Tunis, Tunisia; 6 Glass Department, Institute of Ceramics and Glass (CSIC), Campus Cantoblanco, 28048 Madrid, Spain; Priegopv@icv.csic (F.P.); castro@icv.csic.es (Y.C.); 7 Chemistry Division, CIEMAT, 28040 Madrid, Spain; mariabelen.gomez@ciemat.es; 8 Instituto de Ciencia de Materiales de Madrid-CSIC (ICMM), 28049 Madrid, Spain; cmunuera@icmm.csic.es

**Keywords:** Al doped ZnO-MgO powder, solid-state method, thin films, RF magnetron sputtering, working pressure, optoelectronic properties, photovoltaic applications

## Abstract

In this study, high transparent thin films were prepared by radio frequency (RF) magnetron sputtering from a conventional solid state target based on ZnO:MgO:Al_2_O_3_ (10:2 wt %) material. The films were deposited on glass and silicon substrates at the different working pressures of 0.21, 0.61, 0.83 and 1 Pa, 300 °C and 250 W of power. X-ray diffraction patterns (XRD), atomic force microscopy (AFM), UV-vis absorption and Hall effect measurements were used to evaluate the structural, optical, morphological and electrical properties of thin films as a function of the working pressure. The optical properties of the films, such as the refractive index, the extinction coefficient and the band gap energy were systematically studied. The optical band gap of thin films was estimated from the calculated absorption coefficient. That parameter, ranged from 3.921 to 3.655 eV, was hardly influenced by the working pressure. On the other hand, the lowest resistivity of 8.8 × 10^−2^ Ω cm^−1^ was achieved by the sample deposited at the lowest working pressure of 0.21 Pa. This film exhibited the best optoelectronic properties. All these data revealed that the prepared thin layers would offer a good capability to be used in photovoltaic applications.

## 1. Introduction

Recently, transparent conducting oxide (TCO) materials are gaining much attention due to their physical properties. They are very promising for commercial applications such as displays, photovoltaic cells and light emitting diodes [1,2,3,4]. Among TCO materials, zinc oxide (ZnO) is one of the most used because of its relatively large band gap energy of 3.3 eV at room temperature, large exciton binding energy of 60 meV [5], high optical transmittance of ≥80% and Hall mobility at around 200 cm^2^/Vs at room temperature [6]. In addition, ZnO also presents low cost, high chemical, thermal and mechanical stability [7].

However, the relatively high electrical resistivity of ZnO makes it unsuitable for several applications, and because of that, the doping with different elements to improve the resistivity is studied [8,9]. Most of these studies are focused on the use of a single doping element [10] to improve the ZnO optoelectronic properties. In this sense, highly n-type doped ZnO can be prepared by doping with the group IIIB elements B, Al, Ga, and In, which act as shallow donors. ZnO can be also alloy with II-VI compounds semiconductors such as MgO to increase its band gap. Mg^2+^ shows the particular feature of having its ionic radii very close to Zn^2+^. In addition, co-doping is another alternative that is considered as an effective method to enhance the physical and optical properties of ZnO. Traditionally the co-doping in ZnO thin films has been prepared by using the combination of the group III and group VI elements. However, the combination between Al-Mg has not been explored a lot yet and it could be very advantageous to open new applications in electronic or optoelectronic fields. Several deposition techniques have been used to fabricate ZnO thin films. Among them, one of the most interesting is the magnetron sputtering because of its great advantages of low cost, simplicity, reproducibility, good adhesion, large area substrates, and its proximity to industrial manufacture. However, the raised prices of commercial targets based on co-doped ZnO make them almost prohibitive.

Under this framework, this work presents the fabrication of targets based on the ZnO matrix and co-doping with MgO and Al_2_O_3_ compounds using a low-cost fabrication method as it is the solid-state-method. The main aim is to exploit the target as a cost-effective source for the fabrication of thin films by radio frequency (RF) magnetron sputtering. The choice of the dopant compounds is based on the close atomic radius (1.82 Å for Al and 1.72 Å for Mg) to that of Zn (1.53 Å). The simple doping with Mg provides the widening of the band gap energy of ZnO, as it was demonstrated in our previous work with Mg-doped ZnO NCs fabricated by sol-gel method [11]. Adding an important amount of Mg may lead to the formation of ZnO-ZnMgO-MgO nanocomposites with large band gap energy [12], which would permit to take advantage of the whole spectral range. On the other hand, the introduction of Mg into ZnO lattice does not enhance the additional carrier but, it diminishes the carrier mobility and improves the scattering centers [13]. For this reason, doping with a donor provides the formation of more desirable component for the applications in the field of optoelectronic devices. Hence, Al is the most common donor to obtain n-type ZnO [14,15,16,17]. These III-VI material compounds can show high UV (ultraviolet) luminescence, and thus, they can be used as active transparent materials in the UV region of the spectrum, very useful for optoelectronic applications thanks to their conductivity enhancement [18]. On the other hand, these films present high energy band gap values, and hence, they are also used as CdS substitute in thin film based solar technologies such as those based on antimony sulfide, antimony sulfide selenide or copper zinc selenate materials [19,20].

Therefore, the main aim of this work is devoted to the fabrication of targets by the conventional solid state method from ZnO, MgO and Al_2_O_3_ powders to obtain Mg:Al (10:2 wt %) co-doped ZnO targets with relatively high MgO content of 10 wt %. The optoelectronic performance of Al doped ZnO-MgO (AMZO) thin films deposited from the low-cost target by radio frequency (RF) magnetron sputtering are also investigated. In addition, the effects of working pressure on structural, optical and electrical properties were examined systematically. This work demonstrates the possibility of fabricating mechanically stable and reproducible home-made targets, with diameters as large as 4-inches, that present enough quality to achieve thin films with appropriate properties to be used in optoelectronic devices.

## 2. Materials and Methods

The 4-inch size diameter ceramic targets were fabricated by the conventional solid-state reaction method from a mixture of 98% pure ZnO, 97% pure MgO and 99% pure Al_2_O_3_ powders, respectively. The powders were very well crushed and then blended using a mixer for 30 min to achieve a target with good homogeneity. For 3 h, the mixture was treated at 1050 °C. After that, it was crushed, sifted and mixed in the blender. Then, 4% ethanol of the weight of the powder was added to make it more agglomerated. Finally, the powder was pressed using a cold isostatic press with 3820 Pa, and then sintered during 2 h at 1050 °C. Both steps were crucial to make a hard pellet and to evaporate the added amount of ethanol from which mechanically stable targets were obtained.

The AMZO thin films (CIEMAT, Madrid, Spain) were deposited on Corning 7059 glass (Corning Inc., Corning, NY, USA) and silicon substrates using UNIVEX 450B (Leybold, Cologne, Germany) magnetron sputtering system with confocal geometry. The target’s choice was carried out according to the best structural properties: that one based on ZnO powder with 10 moL.% of MgO and 2 moL.% of Al_2_O_3_. This choice is based on the atomic radii of both dopants to avoid distortions into the lattice and also the possible formation of impurities in the target material during the fabrication process, taking the size of the target into account. Hence, the amount of Mg is high enough to achieve an increase in the band gap energy of the thin films, and the corresponding amount of Al, with higher atomic radii, is the minimum to reach electrical conductivity in the films. Before the deposition process, the substrate was cleaned with isopropyl alcohol and dried by blowing nitrogen over it. The sputtering chamber was evacuated to 10^−5^ Pa and after that, the sputtering process was carried out at different working pressures ranged from 0.21 to 0.82 Pa. The argon (Ar), with a purity of 99.999%, was used as working gas, and its flux was controlled by a mass flow controller. The RF power and the substrate temperature were fixed at 250 W and 300 °C, respectively. Deposition rate was estimated to be ranged from 0.052 nm/s at 0.21 Pa to 0.42 nm/s at 1 Pa, while the deposition time was fit to a layer thickness of 100 nm.

The phase structure of the fabricated target was performed with XRD measurements using Philips X’Pert diffractometer (Malvern, UK) operating in θ-θ configuration with CuKα radiation (45 kV—40 mA) in the angular range of 20° < 2θ < 60°. Phase identification was obtained by comparison with ICS Database.

On the other hand, the crystalline quality and the orientation of the AMZO thin films are evaluated by X-ray diffraction method using Philips X’Pert diffractometer (Δ2θ = 3–70°, 0.017° as increment) supplied with copper X-ray tube (λ = 1.5406 Å), at 40 kV and 100 mA.

The morphology of the film surface was observed with an AFM system with commercial Nano-sensors tips and controlled by software from Nanotech. Topographic images of 2 × 2 µm^2^ areas were acquired in dynamic mode exciting the tip at its resonance frequency of 75 kHz.

Optical reflectance and transmittance measurements were carried out with a PerkinElmer Lambda 1050 UV/Visible/NIR spectrophotometer (Waltham, MA, USA) with a module of three detectors for testing (PbS, InGaAs and photomultiplier tube, PMT, Waltham, MA, USA). The measurements were performed with an interval of 4 nm in the wavelength range of 250–2500 nm, at normal incidence and RT. As reference for reflectance measurements, an aluminum mirror was used. Finally, the electrical properties of the thin films were tested by an ECOPIA HMS-3000 Hall (Tunis, Tunisia) effect measurements system in the van der Pauw configuration with a 0.51 T magnet. The samples were cut into the square size of about 0.5 × 0.5 cm^2^. Ohmic contacts were performed on the four corners of the samples using small indium blobs. The system includes a software with I-V curve capability in order to check the ohmic integrity before making the Hall effect measurements.

## 3. Results

### 3.1. Structural Properties of the ZnO-MgO:Al_2_O_3_ (10:2 wt %) Target

**Figure 1:**
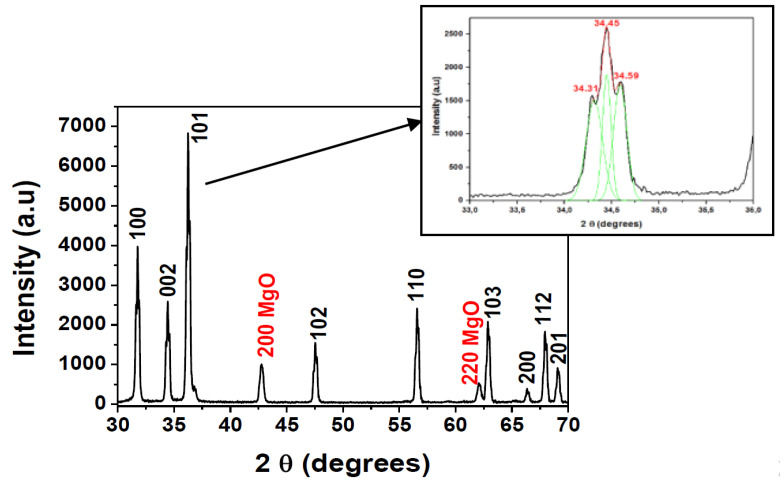
X-ray diffraction patterns of ZnO-MgO:Al_2_O_3_ (10:2 wt %) target annealed and sintered at 1050 °C.

The XRD pattern of the home-made target is pictured in Figure 1. It can be appreciated that the main diffraction peaks of ZnO located at around 2θ = 31.79°, 34.45°, 36.30°, 47.61°, 56.65°, 62.87°, 66.39°, 67.98°, 69.11° corresponded to the (100), (002), (101), (102), (110), (103), (200), (112), (201) planes of the hexagonal würtzite structure of ZnO, respectively. Two extra peaks observed at the positions between 42.79° and 62.11° were relative to the (200) and (220) planes of cubic MgO phase [12].

The preferential orientation was (101), and the (002) reflection can be fitted onto three Gaussian peaks, as shown in the inset of Figure 1. It can be observed three peaks with maxima at 2θ = 34.31°; 34.45° and 34.59°. The main peak at 34.45° was related to the (002) reflection, while the other two were assigned to ZnMgO phase, as demonstrated previous works with samples elaborated by sol-gel method [12,21]. Extra peaks related to Al_2_O_3_ or MgAl_2_O_4_ phases were not detected.

The crystallite size was estimated using the FWHM of the preferred orientation, (101) peak, using the Scherrer formula [21]:

(1) $$D=\frac{K\lambda }{\beta \cos\left(\theta \right)}$$

where K is the Scherrer constant with a value of 0.9, λ is the wavelength of incident radiation, β is the full width at half maximum (FWHM) and θ is Bragg’s angle. The average lattice strain ε of the prepared target is obtained from the following expression:

(2) $$\varepsilon \;=\frac{\beta }{4\tan\theta }$$

Finally, the dislocation density is calculated using [22]:

(3) δ=1D2

Both parameters notify about the magnitude of defects in the crystal.

Table 1 shows the comparison of the structural parameters of the ZnO target doped with different amount of MgO, specifically with 2 wt % and 10 wt %, and the ZnO target co-doped with MgO and Al_2_O_3_(10 wt % and 2 wt %, respectively). As it can be appreciated, when Al_2_O_3_ powder was incorporated in the mixture, a shift of the (101) peak position to lower 2-theta value, smaller nanoparticle size, higher lattice distortion and a wider FHWM were observed. Those phenomena may be related with the elongation suffered in the crystal structure due to the incorporation of Al^3+^ with higher ionic radii than Mg^2+^; and also by the introduction of the important amount of MgO of 10 wt % and formation of ZnMgO phase that could lead to an increase of defects and disorder into the ZnO lattice [23,24]. 

In this work, the ZnO-MgO:Al_2_O_3_ (10:2 wt %) target was used to deposit thin films to take advantage of the ZnMgO phase appearance for solar cells applications.

### 3.2. Structural Properties of AMZO Thin Films

**Figure 2:**
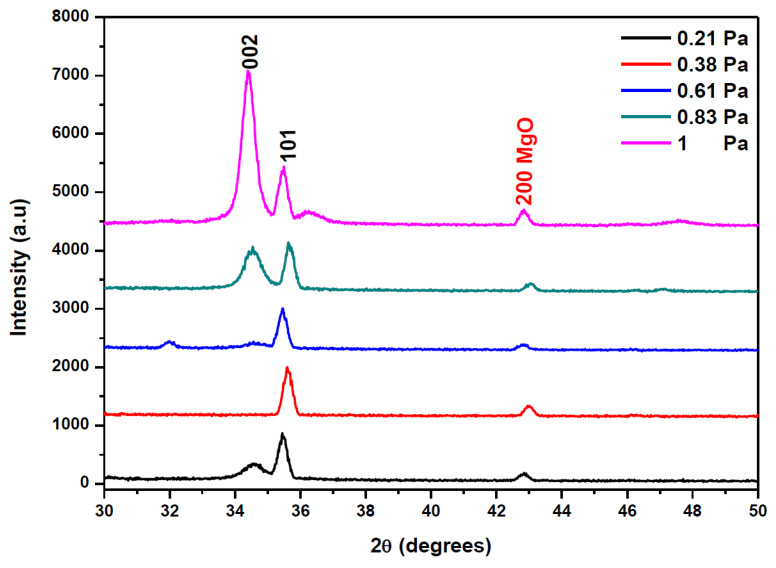
XRD patterns of AMZO thin films deposited at different working pressure values.

The XRD spectra of AMZO thin films deposited at 300 °C at different working pressures are shown in Figure 2.

All AMZO thin films presented hexagonal wurtzite structure with (101) as a preferred orientation. However, the increase of the sputtering pressure evolved the polycrystalline structure of ZnO. The (002) reflection peak appears as a preferred orientation at the highest working pressure of 1 Pa. This explains the tendency of a change in the grain orientation towards an ordered structure at those deposition conditions. Several studies about Mg and Al co-doping ZnO thin films fabricated by RF magnetron sputtering consider the (100) direction as the preferred orientation [24], but a change can occur due to the large density of target impurities [25]. In addition, a small extra (200) peak related to MgO phase was observed. The appearance of this peak was explained because of the high concentration of MgO in the target. No phases corresponding to ZnAl_2_O_3_ and Al_2_O_3_ indicating the effective substitution of Zn^2+^ with Al^3+^ ions. It seems that the increase of the working pressure did not affect significantly the position of diffraction peaks but rather their intensity. The influence of working pressure was also investigated by the evaluation of the average crystallite size calculated from (002) and (101) reflection peaks using the Scherrer Formula (1). The results show that crystallite size varied from 26 to 28 nm as function of the working pressure, as it is summarized in Table 1. The strain ε was kept nearly to 0.004 for all samples and hence, it was not influenced by the increase of the working pressure. In addition, Table 1 listed the calculation of the residual stress σ in AMZO thin films, by using the following formula [26]:

(4) $$\sigma =\left[2{\;C}_{13}-\frac{C_{33}\left(C_{11}+C_{12}\right)}{C_{13}}\right]\left[\frac{C-{\;C}_{0}}{C_{0}}\right]$$

where C_ij_ are elastic stiffness constants, C_13_ = 1.05 × 10^11^ Pa, C_33_ = 2.1 × 10^11^ Pa, C_11_ = 2.1 × 10^11^ Pa, C_12_ = 1.2 × 10^11^ Pa [27], C is the lattice parameter and C_0_ is the bulk value, 5.206 Å. The numerical calculation leads to this summary expression:

(5) $$\sigma =-4.5\times 10^{11}\left[\frac{C-{\;C}_{0}}{C_{0}}\right]\frac{N}{m^{2}}$$

The obtained values did not depend on the working pressure and they were in the order of 4.495 10^11^ Pa for all samples, as it was expected. The films deposited at working pressures below 0.61 Pa exhibited the (101) as a preferred orientation, identical to what was shown in the spectrum of the nano powders (see Figure 1). In addition, the intensity of (101) peak was higher than (002) peak, almost negligible at that pressure range, suggesting that the surface energy of (101) was the lowest at those sputtering conditions. However, at working pressures of 0.83 Pa, the (002) peak began to appear with the same intensity that the (101) peak. At the highest working pressure of 1 Pa, the (002) crystallographic direction became the preferential orientation, indicating that the preferential orientation of the crystallites was perpendicular to the film surface. It is believed that this change in the preferred orientation was a consequence of a self-ordering caused by the minimization of the crystal surface energy [28], and by the change in diffusion rate of atoms at the surface during the deposition process resulting on the increase of the working pressure [29].

### 3.3. Surface Morphological Analysis

**Figure 3:**
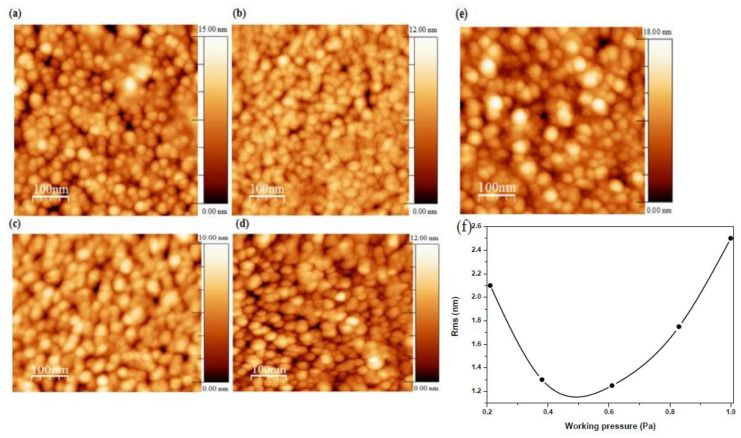
Micrographs of AMZO thin films deposited at different working pressure values of (**a**) 0.21 Pa, (**b**) 0.38 Pa, (**c**) 0.61 Pa, (**d**) 0.83 Pa, (**e**) 1 Pa from the ZnO-MgO:Al_2_O_3_ (10:2 wt %) target, and (**f**) the evolution of RMS as a function of working pressure.

AFM images of AMZO thin films deposited at 300 °C and at different working pressures are shown in Figure 3. Grains with a feature size of 22–30 nm were presented in the films, similar to those values calculated from XRD spectra (see Table 1). Spherical, uniform and dense grains were also produced throughout the surface.

The sample deposited at the lowest working pressure of 0.21 Pa presented some voids, which were reduced with the working pressure. Consequently, the root-mean-square (RMS) roughness was decreased from 2.1 to 1.3 nm as working pressure increased up to 0.6 Pa attributed to the slightly smaller crystallite size. For working pressures above this value, the RMS increased quickly up to 2.5 nm, in agreement with the observed enhancement of the c-axis orientation for the sample deposited at 1 Pa. The more the working pressure increased, the higher the number of Zn atoms were substituted by dopant elements. This fact favored the increase of the intensity of (002) orientation, leading to a better structural ordering. This hypothesis was also confirmed by other authors using different dopants that also observed an increase of the roughness [30,31]. In addition, the increase of surface roughness endorsed oxygen absorption on the crystallites surface’s which created dangling bonds performing as electron traps. These electron traps would be the responsible on the reduction in carrier concentration [32] behavior that will be discussed later.

### 3.4. Optical Properties

**Figure 4:**
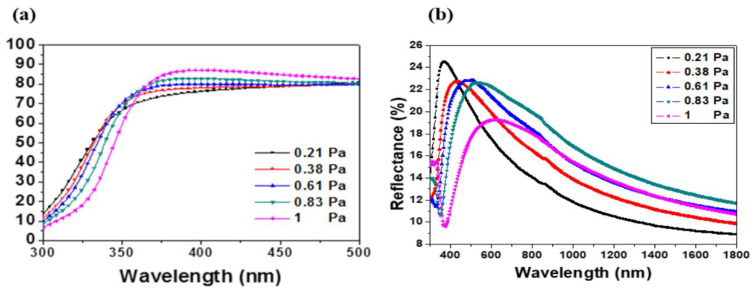
(**a**) Transmittance and (**b**) reflectance spectra of AMZO thin films prepared at different working pressures.

The transmittance and reflectance spectra of the sputtered films are pictured in Figure 4a,b. As it can be appreciated, the films were highly transparent in the visible wavelength range. The transmittance T% was close to 80% and it was enhanced with the working pressure.

The absorption edge in the ultraviolet range was investigated by the evaluation of the band gap of the films. As it can be observed in Figure 4, the UV absorption edge moved toward the shorter wavelengths, which may be related to the variation of residual stress and crystal grain size of the films. The band gap was estimated by the absorption coefficient with respect to the incident photon energy [33] as followed:

(6) $${\left(\alpha h\upsilon \right)}^{2}=A\left(h\upsilon -E_{g}\right)$$

where, A is a constant, hυ is the photon energy, E_g_ is the band gap energy and α is the absorption coefficient.

**Figure 5:**
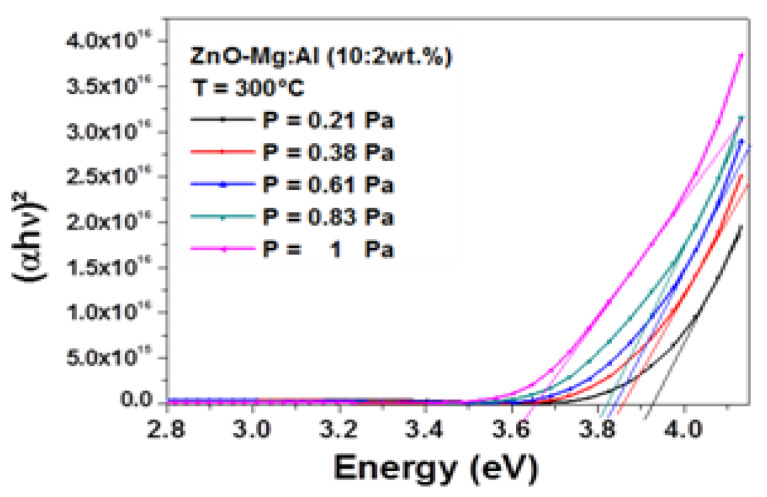
Spectra of (αhυ)^2^ vs. photon energy.

Figure 5 shows the Tauc plot as function of the working pressure. The band gap was obtained by extrapolating the linear part of the curves to the horizontal axis. A monotonic decline was very well seen as the working pressure rising from 0.21 to 0.83 Pa. A decrease from 3.921 to 3.655 eV with increasing the working pressure from 0.21 to 1 Pa was observed.

On one hand, it can be remarked that these values were larger than the band gap energy of bulk undoped-ZnO, estimated to be 3.37 eV, despite the absence of quantum confinement effect since the crystallite sizes were larger than 10 nm. This blue shift may be attributed to the enhancement of the carrier concentration in ZnO, known as the Burstein–Moss effect [34,35]. In fact, Al_Zn_ donors [36] can be created from the process of Al^3+^ doping. By following the same analysis, the reduction of the band gap energy of AMZO film with the working pressure was attributed to a reduction of carrier concentration. More ionized impurity scattering, impurity clustering and grain boundary that would lead to a loss of free electrons, were certainly created by increasing the working pressure.

The following equation describes the Burstein–Moss effect [37]:

(7) $$\Delta E_{g}=\frac{h^{2}N^{2/3}}{8m_{e}\pi^{2/3}}$$

where h is the Planck constant, N is the carrier concentration, and m_e_ is the effective mass of electrons. From the above equation, the optical band gap is proportional to the carrier concentration. Here, few electrons populating the states near the bottom of the conduction band would explain the reduction of the band gap energy [38].

**Figure 6:**
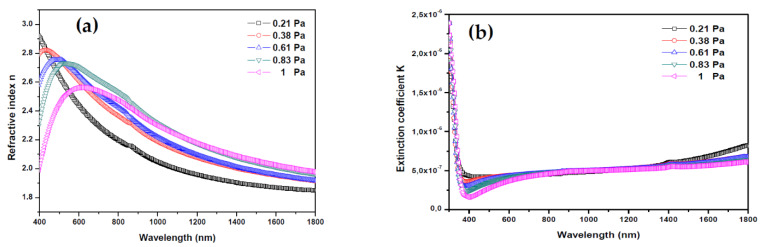
(**a**) Refractive index and (**b**) extinction coefficient of thin films deposited from ZnO-MgO:Al_2_O_3_ (10:2 wt %) target at various working pressures.

Figure 6 shows the evolution of refractive index n and extinction coefficient K of AMZO thin films deposited at different working pressures.

The refractive index is related to the transmittance, reflectance and extinction coefficient K by the following expression [39,40]:

(8) $$n=\frac{\left(1+\;R\right)}{\left(1-R\right)}+\sqrt{\frac{4R}{{\left(1-R\right)}^{2}}-K^{2}}$$

(9) $$K=\frac{\alpha \lambda }{4\pi },$$

where the K values were very low, in the range of 10^−8^, indicating the low dielectric losing in these films. It may be considered a qualitative indication of the excellent smoothness of thin films [41]. The refractive index plotted in Figure 6a showed a decrease in the main peak intensity between 400 and 600 nm with increasing the working pressure. From this peak, the refractive index values were 2.91, 2.82, 2.69, 2.67, and 2.59 for 0.21, 0.38, 0.61, 0.83 and 1 Pa, respectively. In all cases, they were greater than the refractive index of bulk ZnO (2.0) [42]. These relatively high values are preferred for antireflection coating materials in many optoelectronic device applications. As Z.C. Tu et al. [43] reported, the energetic position of the maximum band of the refraction index was directly related with the band gap energy of ZnO. Here, by increasing the working pressure, the band gap energy and the refractive index reduce slightly, which may be due to the formation of impurities and defects.

### 3.5. Electrical Study

The conductivity σ, the Hall mobility μ and the carrier concentration n of AMZO thin films are shown in Table 2.

The carrier concentration n was severely reduced with the working pressure, which is in good agreement with the Burstein–Moss effect adopted to justify the large reduction of the band gap energy E_g_, estimated to be 0.2 eV. It has been reported [44] that the increase of the working pressure induces a reduction of the kinetic energy of the dopant atoms which would be limiting their energy of surface diffusion. Consequently, the activation amount of Al^3+^ and Mg^2+^ dopants would decrease. Thus, the carrier concentration also decreased with the working pressure.

To the contrary, the Hall mobility μ presented the opposite evolution of the carrier concentration n, while the product of μ and n decreased with the working pressure. As consequence, the decrease of the conductivity σ with the working pressure can be attributed since σ is proportional to μ and n. Finally, the high improvement of the mobility was due to the lower carrier concentration and hence the reduction of the number of collisions between the electrons into the grain boundaries. In addition, the loss of kinetic energy was lower, which offered both greater area and energy to the carriers to move freely.

## 4. Conclusions

AMZO thin films were successfully deposited on Corning glass by RF magnetron sputtering from a home-made fabricated 4-inch diameter ZnO-MgO:Al_2_O_3_ (10:2 % wt) target using the conventional solid-state method. Prior to be used in the sputtering system, the mechanical stability and the structural quality of the target to fabricate AMZO thin films were evaluated and demonstrated.

With regards to the AMZO thin films, the effect of the working pressure on their structural, optical and electrical properties was studied. A progressive change of the axis preferred orientation of the growth from (101) to (002) was observed, but no significant change in the crystallite size and the lattice strain was detected. Hence, XRD results put into evidence that increasing the working pressure enhanced the crystalline quality of the films. Optical analysis showed larger values of the band gap energy of the AMZO films compared to that of un-doped ZnO, attributed to the Burstein–Moss effect. However, the reduction of the band gap energy with the working pressure was due to the decreased of carrier concentration, as it was revealed from the Hall measurements. These measurements showed that the increase of working pressure affected the electrical conductivity as a result of the inverse trend of carrier concentration and Hall mobility. Finally, the good optical and electrical performance of the AMZO films presented in this work make them suitable for optoelectronic and photovoltaic applications.

## Figures and Tables

**Table 1:** Structural parameters for the ZnO-based targets fabricated by using the solid-state method and for the AMZO thin films fabricated using the ZnO-MgO:Al_2_O_3_ (10:2 wt %) target.

**ZnO-Based Targets**						
**%wt MgO**	**%wt Al_2_O_3_**	**2 θ of (101) Peak (Degrees)**	**D (nm)**	**δ (nm^−2^)**	**Strain ε**	**FWHM of (101) Peak (Rad)**
**2**	0	36.61	81.0	0.0001	0.0014	0.0018
**10**	0	36.30	29.0	0.0012	0.0039	0.0051
**10**	2	36.25	22.3	0.0020	0.0053	0.0064
**AMZO Thin Films Fabricated from** **ZnO-MgO: Al_2_O_3_ (10:2 wt %) Target**						
**Pressure (Pa)**		**2 θ of (101) Peak (Degrees)**	**D (nm)**			**σ (10^11^ Pa)**
**0.21**		35.45	28.11 ± 2.2			4.4955
**0.38**		35.60	26.92 ± 1.5			4.4953
**0.61**		35.47	27.51 ± 2.0			4.4954
**0.83**		35.64	28.56 ± 2.8			4.4956
**1.00**		35.50	27.55 ± 4.5			4.4954

**Table 2:** Electrical properties of thin films deposited at different working pressures.

Pressure (Pa)	σ (Ω^−1^·cm^−1^)	μ (cm^2^/Vs)	n (10^15^·cm^−3^)
**0.21**	11.25	89.64	783
**0.61**	6.20	135.10	286
**0.83**	2.76	228.90	75
